# Aggressive Subcutaneous Panniculitis-Like CD30+ Peripheral T-Cell Lymphoma with Diffuse EBER Expression

**DOI:** 10.1155/2014/874725

**Published:** 2014-07-20

**Authors:** Amandeep Aneja, Raghava LevakaVeera, Viren Patel, Ashish Bains

**Affiliations:** ^1^Department of Pathology, Temple University Hospital, 3401 N Broad Street, Philadelphia, PA 19140, USA; ^2^Department of Hematology-Oncology, Temple University/Fox Chase Cancer Center, 3400 N Broad Street, Philadelphia, PA 19140, USA

## Abstract

T-cell lineage lymphoma with an intense membranous and paranuclear CD30 expression in the absence of ALK1 raises a differential diagnosis of peripheral T-cell lymphoma (PTCL), NOS and anaplastic large cell lymphoma (ALCL), ALK negative. However, Epstein-Barr virus is consistently negative in ALCL and is not considered an implicating factor in its pathogenesis. We describe a case of T-cell lymphoma showing anaplastic large cell morphology with scattered hallmark cells and a uniform CD30 and Epstein-Barr virus encoded early RNA (EBER) expression that primarily involved the subcutaneous tissue at presentation. On incisional biopsy, the neoplastic cells were positive for CD3, CD2, and CD30 while negative for LCA, CD20, PAX5, CD56, ALK1, and cytotoxic granules. Molecular analysis identified a positive T-cell receptor (beta and gamma) gene rearrangement by PCR. Proliferation index approached 100% and the patient had a rapidly progressive course; the subcutaneous lesions more than doubled in size within couple of weeks with new evidence for widespread systemic involvement. This case emphasizes a rare EBV association with a CD30 positive T-cell lymphoma where the morphologic and immunophenotypic findings are otherwise nondiscriminatory between PTCL, NOS and ALCL, ALK negative.

## 1. Introduction

Mature T-cell lymphomas are diverse group of aggressive neoplasms with immunophenotype that varies greatly from case to case. CD30 expression in a T-cell lineage lymphoma, with intense membranous and paranuclear staining, is characteristically a marker for identifying anaplastic large cell lymphoma (ALCL) [1]. Since the original description of Ki-1 lymphoma [2], B-cell lineage lymphomas have been excluded from the category of ALCL. However, a subset of peripheral T-cell lymphoma, not otherwise specified (PTCL, NOS) displays large-cell morphology with substantial CD30 expression, rendering a precise distinction from ALCL, ALK negative problematic. Cutaneous ALCL is a distinct entity with an absence of ALK expression. Although there are no well-defined criteria to discern a more aggressive systemic involvement from a localized cutaneous form, the latter has a much favorable prognosis. Expansive staging methods are required to exclude a systemic disease before considering a diagnosis of primary cutaneous ALCL.

Herein, we describe a patient with diffusely Epstein-Barr virus (EBV) positive T-cell lymphoma, primarily involving the subcutaneous tissue. The lymphoma had a proliferation index approaching 100% with rapid progression to systemic involvement and more than doubling in size of subcutaneous nodules within couple of weeks from diagnosis. This case emphasizes a peculiar CD30 positive immunophenotype with uniform Epstein-Barr encoded early RNA (EBER) expression in a subcutaneous T-cell lymphoma where the clinical presentation, morphology, and immunophenotype present a diagnostic dilemma between ALCL, ALK negative and PTCL, NOS.

## 2. Case Presentation

**Figure 1:**
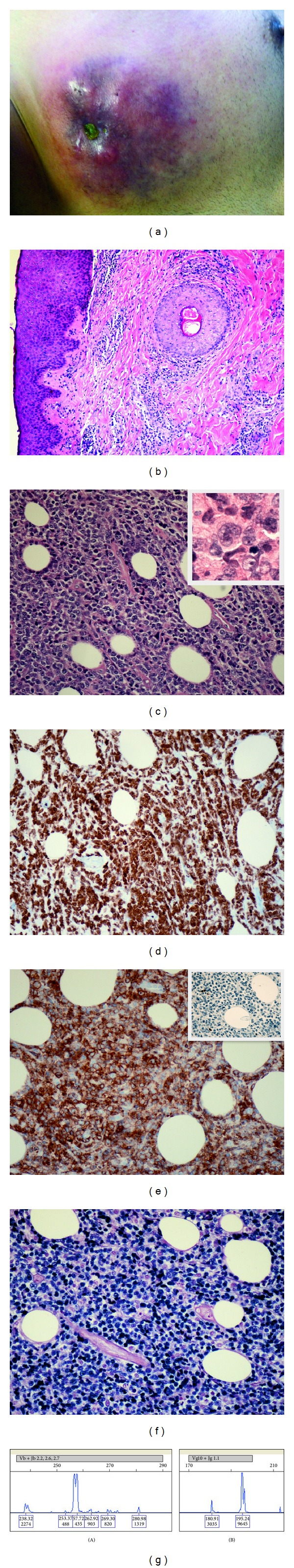
(a) Chest wall subcutaneous mass that rapidly enlarged in size. Epidermal scab is after incisional biopsy. (b) H&E showing uninvolved epidermis (10x). (c) H&E of the lesion with large neoplastic cells rimming the subcutaneous adipose tissue (20x). Inset shows scattered neoplastic cells with “hallmark cell” morphology. (d) and (e) Immunohistochemical stains strongly positive for CD3 and CD30, respectively (20x). Inset (e) shows a negative TIA1 stain in tumor cells with scattered positive cells corresponding to reactive CD8+ T-cells. (f) In-situ hybridization for Epstein-Barr virus encoded early RNA with diffuse strong expression in the tumor cells. Endothelial cells on a blood vessel seen here are negative (20x). (g) Positive T-cell receptor gene rearrangement: (A) TCR-*β* and (B) TCR-*γ*.

A 42-year-old Hispanic man presented with painful multiple subcutaneous soft tissue nodules on neck, trunk, and left upper extremity. Few weeks prior to presentation, he noted small papule on left anterior chest wall that rapidly progressed to larger tender mass. Meanwhile, four new similar masses surfaced on his trunk. At presentation the masses were firm with restricted mobility and ranged from 3 to 5 cm in largest dimension.  Figure 1(a) shows the left chest wall mass approximately 3 weeks after incisional biopsy. The patient had no significant past medical or surgical history. Computed tomography (CT) scan showed infiltrative subcutaneous tissue masses without other sites of involvement or lymphadenopathy. CBC showed normal indices (8,600/*μ*L WBC count with normal differential, 13.3 gm/dL hemoglobin, and 234 K/*μ*L platelets). Basic metabolic profile, hepatic function panel, and coagulation tests were normal. Lactate dehydrogenase and uric acid levels were elevated at 2413 U/L and 8.6 mg/dL, respectively. HIV and hepatitis test panels were negative.

Biopsy from the left chest wall and right abdominal nodules showed skin and subcutaneous tissue with extensive neoplastic lymphocytic infiltrate. The infiltrate mainly involved the subcutaneous tissue with focal dermal extension and sparing of the epidermis [Figures 1(b) and 1(c)]. Neoplastic cells morphologically ranged in spectrum from medium to large to anaplastic with marked nuclear irregularities. Moderately abundant cytoplasm and scattered hallmark cells were identified [Figure 1(c) inset]. Immunohistochemical studies performed with appropriate controls revealed that the neoplastic cells were positive for CD2, CD3 (Figure 1(d)), and CD30 (Figure 1(e)), while negative for leukocyte common antigen (CD45), CD4, CD5, CD7, CD8, CD20, PAX5, CD56, TIA1, Granzyme B, ALK1, and Beta-F1. In-situ hybridization established a diffuse expression of EBER [Figure 1(f)]. A clonal T-cell receptor gene rearrangement was identified by PCR involving both beta and gamma genes [Figure 1(g)]. An interval increase in the size of subcutaneous lesions with development of new subcutaneous masses and left inguinal lymphadenopathy was clinically identified over the next few weeks. Staging PET CT scan at three weeks from diagnosis showed highly metabolic 18F-FDG active (SUV range 6.2–30) subcutaneous masses, multiple peripheral and mesenteric lymph nodes, and left adrenal gland. Bone marrow biopsy was negative for lymphomatous involvement. Based on overall findings, a diagnosis of CD30 and EBV positive T-cell lymphoma primarily involving the subcutaneous tissue was confirmed which could be best classified as PTCL, NOS in terms of the absence of TIA1 and granzyme B expression and the presence of EBV infection.

## 3. Discussion

T-cell lymphomas primarily involving the skin and subcutaneous tissue are a heterogeneous group of neoplasms with diverse clinical features and prognosis. According to the World Health Organization (WHO) classification, distinct clinicopathological entities with stringent diagnostic criteria have been described. However, several mature T-cell lymphomas do not belong to any of the defined classification category and are listed as PTCL, NOS. These tumors account for approximately 26% of mature T-cell lymphoma, with skin and GI tract representing the most common extranodal affected sites [3].

In this reported case, a homogeneously strong CD30 expression with negative cytotoxic markers and positive EBER-ISH is an unusual immunophenotype for a T-cell lymphoma. As currently defined, ALCL, ALK negative comprises CD30(+) T-cell neoplasms that are not reproducibly distinguishable on morphological grounds from ALCL, ALK positive. However, the former lacks ALK gene rearrangement and protein expression with most cases expressing T-cell-associated markers and cytotoxic granule-associated proteins [3]. Further, WHO states that ALCL is “consistently negative for Epstein-Barr virus” and studies have postulated that there is no role of EBV infection in ALCL [4]. Contrary to this belief, the neoplastic cells in this case were diffusely positive for EBV (EBER-ISH) and negative for cytotoxic markers. Although ALCL can be negative for cytotoxic marker in a minority of cases, overall findings with uniform EBER expression in our case hinder a definite classification as ALCL, ALK negative under the current schema. Recent reports, however, have suggested that presence of EBV does not categorically exclude a diagnosis of ALCL, ALK negative [5]. The earlier belief, that ALCL, ALK negative does not seem to be distinctive at the immunophenotypic or molecular level and the prognosis is similar to that of patients with PTCL, NOS, has been challenged by clinical and gene expression profiling data [6]. These studies support their existence as two separate disease entities; nonetheless, the border between ALCL, ALK negative and PTCL, NOS is a matter of conjecture and imprecise.

Conflicting survival data are available today with regard to ALCL, ALK negative and PTCL, NOS. Berge et al. [7] reported a comparable poor prognosis for these two entities (5-year overall survival: <45%), proposing that the segregation of the two entities might be of limited clinical relevance. Peripheral T-cell lymphoma project [8] on the other hand highlighted the outcome differences between PTCL, NOS and ALCL, ALK negative in which 5-year FFS (36% versus 20%) and OS (49% versus 32%) were superior in ALCL, ALK negative compared to PTCL, NOS. Bisig et al. [9] compared the expression profiles of 16 PTCL, NOS [six CD30(+) and ten CD30(−)] and 35 ALCL [25 ALK(+) and 10 ALK(−)] in their study of CD30(+) peripheral T-cell lymphomas and showed that their molecular findings further corroborated the biological continuum across CD30(+) PTCL. They concluded that the distinction from CD30(+) PTCL, NOS, particularly from those cases composed of large pleomorphic cells, may be fragile and subjective. CD30 expression may be significantly higher in the presence of Epstein-Barr virus (EBV). In a study by Smuk et al. [10], 10 of 11 cases of EBV-associated PTCL-NOS were found to express CD30. The prognostic significance of presence of EBER in the tumor still remains to be established. Dupuis et al. [11] have shown that the presence of EBV-encoded RNA in the tumor tissue is an adverse predictor of survival in older patients (>60 years) with PTCL-NOS. In contrast, peripheral T-cell lymphoma project found that significant EBV infection (EBER 3 to 4+) was predictive of poor survival only in younger patients (<60 years).

When faced with an EBV(+) T-cell lymphoma, extranodal NK/T-cell lymphoma, nasal type is also a diagnostic consideration. Patient's ethnicity and the expression of CD2 in this case would support that possibility. Though clonal T-cell receptor gene rearrangement has been reported in 7–38% of extranodal NK/T-cell lymphoma cases [12], in the absence of CD56, CD8, TIA1, and granzyme B made extranodal NK/T-cell lymphoma, nasal type an unlikely diagnosis. We felt that this case was best categorized as a CD30 and EBV positive T-cell lymphoma lacking a more precise and definite classification. However, we propose that ALCL, ALK negative in the presence of EBV cannot be entirely excluded. The specific CD30 expression on the lymphoma cells makes it an attractive target for drug-conjugated antibody-directed therapies, as first reported by Falini et al. [13] which would further justify to maintain this distinction ALCL, ALK negative from PTCL, NOS for innovative and targeted therapeutic approaches. Bisig et al. [9] also suggested that the expression of CD30 might constitute a valuable criterion to define two distinct biological subgroups [CD30(+) and CD30(−)] within the heterogeneous category of PTCL, NOS with the potential benefits of incorporating anti-CD30 immunoconjugates into the treatment strategies of CD30(+) PTCL.

## 4. Conclusion

We hereby report a unique case of CD30 positive T-cell lymphoma with anaplastic large cell morphology and diffusely strong Epstein-Barr encoded early RNA expression which highlights the diagnostic dilemma between ALCL, ALK negative and PTCL, NOS. Under current WHO classification, this case is best classified as PTCL, NOS based on the presented immunophenotype, the absence of TIA1 and granzyme B expression, and the presence of EBV infection. However, significant differences in prognosis and potential for targeted therapeutic approaches emphasize the importance of better defining the diagnostic criteria for an accurate distinction between these two entities.

## References

[1] Medeiros LJ, Elenitoba-Johnson KSJ (2007). Anaplastic large cell lymphoma. *American Journal of Clinical Pathology*.

[2] Stein H, Mason DY, Gerdes J (1985). The expression of the Hodgkin’s disease associated antigen Ki-1 in reactive and neoplastic lymphoid tissue: evidence that Reed-Sternberg cells and histiocytic malignancies are derived from activated lymphoid cells. *Blood*.

[3] Swerdlow SH, Campo E, Harris NL (2008). *WHO Classification of Tumours of Haematopoietic and Lymphoid Tissues*.

[4] Herling M, Rassidakis GZ, Jones D, Schmitt-Graeff A, Sarris AH, Medeiros LJ (2004). Absence of Epstein-Barr virus in anaplastic large cell lymphoma: a study of 64 cases classified according to World Health Organization criteria. *Human Pathology*.

[5] Ma L, Katz Y, Sharan KP, Schwarting R, Kim AS (2011). Epstein-Barr virus positive anaplastic large cell lymphoma: myth or reality?. *International Journal of Clinical and Experimental Pathology*.

[6] Piccaluga PP, Agostinelli C, Califano A (2007). Gene expression analysis of peripheral T cell lymphoma, unspecified, reveals distinct profiles and new potential therapeutic targets. *The Journal of Clinical Investigation*.

[7] Berge RL, De Bruin PC, Oudejans JJ, Ossenkoppele GJ, Van Der Valk P, Meijer CJLM (2003). ALK-negative anaplastic large-cell lymphoma demonstrates similar poor prognosis to peripheral T-cell lymphoma, unspecified. *Histopathology*.

[8] Savage KJ, Harris NL, Vose JM (2008). ALK—anaplastic large-cell lymphoma is clinically and immunophenotypically different from both ALK + ALCL and peripheral T-cell lymphoma, not otherwise specified: report from the International Peripheral T-Cell Lymphoma Project. *Blood*.

[9] Bisig B, de Reyniès A, Bonnet C (2013). CD30-positive peripheral T-cell lymphomas share molecular and phenotypic features. *Haematologica*.

[10] Smuk G, Illés Á, Keresztes K (2010). Pheno- and genotypic features of epstein-barr virus associated B-cell lymphoproliferations in peripheral T-cell lymphomas. *Pathology and Oncology Research*.

[11] Dupuis J, Emile J, Mounier N (2006). Prognostic significance of Epstein-Barr virus in nodal peripheral T-cell lymphoma, unspecified: a groupe d’Etude des Lymphomes de l’Adulte (GELA) study. *Blood*.

[12] Gualco G, Domeny-Duarte P, Chioato L, Barber G, Natkunam Y, Bacchi CE (2011). Clinicopathologic and molecular features of 122 Brazilian cases of nodal and extranodal NK/T-cell lymphoma, nasal type, with EBV subtyping analysis. *The American Journal of Surgical Pathology*.

[13] Falini B, Bolognesi A, Flenghi L (1992). Response of refractory Hodgkin’s disease to monoclonal anti-CD30 immunotoxin. *The Lancet*.

